# Zika Virus Seroprevalence, French Polynesia, 2014–2015

**DOI:** 10.3201/eid2304.161549

**Published:** 2017-04

**Authors:** Maite Aubry, Anita Teissier, Michael Huart, Sébastien Merceron, Jessica Vanhomwegen, Claudine Roche, Anne-Laure Vial, Sylvianne Teururai, Sébastien Sicard, Sylvie Paulous, Philippe Desprès, Jean-Claude Manuguerra, Henri-Pierre Mallet, Didier Musso, Xavier Deparis, Van-Mai Cao-Lormeau

**Affiliations:** Institut Louis Malardé, Papeete, Tahiti, French Polynesia (M. Aubry, A. Teissier, C. Roche, S. Teururai, D. Musso, V.-M. Cao-Lormeau);; Centre d’Épidémiologie et de Santé Publique des Armées, Marseille, France (M. Huart, S. Sicard, X. Deparis);; Unité Mixte de Recherche Sciences Economiques et Sociales de la Santé et Traitement de l'Information Médicale, Marseille (M. Huart, S. Sicard, X. Deparis);; Institut de la Statistique de la Polynésie Française, Papeete (S. Merceron);; Institut Pasteur, Paris, France (J. Vanhomwegen, S. Paulous, J.-C. Manuguerra);; Direction Départementale de la Cohésion Sociale et de la Protection des Populations, Yonne, France (A.-L. Vial);; Direction de la Santé de la Polynésie Française, Papeete (A.-L. Vial, H.-P. Mallet);; Université de La Réunion and UMR PIMIT, Sainte Clotilde, France (P. Desprès)

**Keywords:** Zika virus, Zika, French Polynesia, flavivirus, arbovirus, seroprevalence, seropositivity, immunoglobulin G, IgG, viruses, vector-borne infections, zoonoses

## Abstract

During 2013–2014, French Polynesia experienced an outbreak of Zika virus infection. Serosurveys conducted at the end of the outbreak and 18 months later showed lower than expected disease prevalence rates (49%) and asymptomatic:symptomatic case ratios (1:1) in the general population but significantly different prevalence rates (66%) and asymptomatic:symptomatic ratios (1:2) in schoolchildren.

Zika virus (family *Flaviviridae,* genus *Flavivirus*), an arthropodborne pathogen, is transmitted to humans by *Aedes* spp. mosquitoes, but nonvectorborne transmission (i.e., maternofetal and sexual transmission and transmission via blood transfusion) has also been reported (*1*). Infection by Zika virus most commonly causes mild disease consisting of fever, rash, arthralgia, headache, and conjunctivitis (*1*), but severe neurologic complications, such as Guillain-Barré syndrome in adults (*2*) and microcephaly in fetuses and newborns (*3*), have been described.

Zika virus emerged for the first time in 2007 on Yap Island, Federated States of Micronesia, in the Pacific region (*4*). Six years later, Zika virus caused an explosive outbreak in French Polynesia (*5*), and the virus then spread across the Pacific region (*6*). During October 2013–April 2014 in French Polynesia, an estimated 32,000 persons (11.5% of the population) visited healthcare facilities because of clinical symptoms suggestive of Zika virus infection (*1*,*7*). A retrospective serosurvey conducted on blood collected from donors before the outbreak confirmed that Zika virus had not previously circulated in French Polynesia (*8*). We conducted a study to assess Zika virus seroprevalence among the French Polynesia population after the virus emerged in the country.

## The Study

French Polynesia comprises 119 islands distributed among 5 archipelagos (Society, Tuamotu, Marquesas, Australs, and Gambier). The population of ≈270,000 inhabitants lives on 74 islands (2012 census; http://www.ispf.pf/docs/default-source/publi-pr/POP_LEGALE_2012_PF.pdf?sfvrsn=2). During February and March 2014, we conducted a cluster sampling among the general population living in the 5 archipelagos. We randomly recruited a total of 196 participants on the most inhabited islands of each archipelago: Tahiti and Moorea (Society), Rangiroa and Makemo (Tuamotu), Nuku Hiva and Hiva Oa (Marquesas), Rurutu (Australs), and Rikitea (Gambier) (Figure). Because >85% of the inhabitants of French Polynesia live on the Society Islands, we conducted a second cluster sampling among 700 participants recruited on Tahiti and Moorea during September–November 2015. In addition, 476 schoolchildren initially recruited for a dengue serosurvey on Tahiti during May and June 2014 were included in the study.

**Figure:**
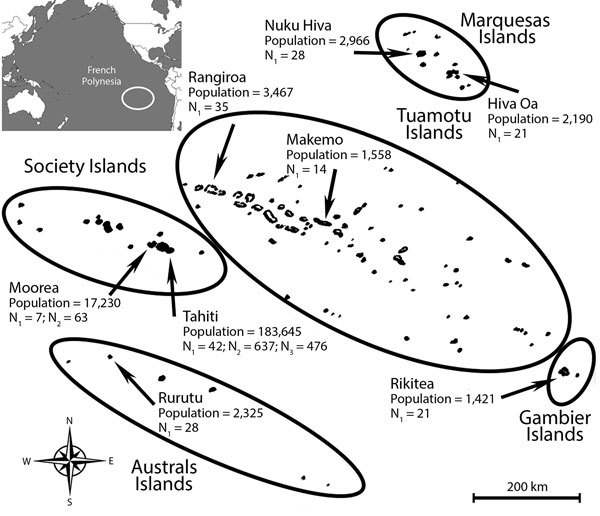
Geographic distribution of participants recruited for a study of the postemergence seroprevalence of Zika virus infections in French Polynesia, 2014–2015. N_1_ and N_2_ indicate areas of recruitment among the general population during February and March 2014 and September–November 2015, respectively; N_3_ indicates areas of recruitment among schoolchildren during May and June 2014. The total population and number of recruited participants is shown for each area. Lines delineate the 5 archipelagos that comprise French Polynesia (Marquesas, Society, Tuamotu, Gambier, and Australs). Inset map at upper left shows location of French Polynesia in the Pacific Ocean (white circle).

All participants were asked to declare whether they had clinical manifestations suggestive of past Zika infection. Adults provided written informed consent before enrollment, and parents or guardians gave consent for their children. Participants’ blood samples and personal data were anonymized before processing, and the study was approved by the Ethics Committee of French Polynesia (no. 60/CEPF 06/27/2013).

We used a recombinant antigen–based indirect ELISA to detect Zika virus IgG in blood samples collected in 2014 from the general population and schoolchildren (*8*). We also tested serum samples from the general population by microsphere immunoassay (MIA), using the same recombinant antigens as for the ELISA (*2*,*8*). Among the 196 serum samples from the general population, 80% tested positive for Zika virus IgG by both assays (κ = 0.51, indicating good agreement between ELISA and MIA results). Blood samples collected in 2015 were tested by MIA only.

Zika virus seroprevalence rates and proportions of asymptomatic infections were 49% (95% CI 42%–57%) and 43% (95% CI 33%–53%), respectively, for participants from the general population sampled in 2014 and 66% (95% CI 60%–71%) and 29% (95% CI 24%–34%), respectively, for schoolchildren sampled in 2014 (Table). Seroprevalence rates and proportions of asymptomatic infections were 22% (95% CI 16%–28%) and 53% (95% CI 45%–61%), respectively, for participants recruited in 2015 (Table).

**Table:** Zika virus seroprevalence among persons randomly recruited from the general population and among schoolchildren immediately after and 18 months after a Zika outbreak, French Polynesia, 2014 and 2015*

Sampled population, time of sampling, location of sampling	Median age (range), y	No. symptomatic/no. positive (% [95% CI])	No. asymptomatic/no. positive (% [95% CI])	Total no. seropositive/total no. tested (% [95% CI])
General population				
February–March 2014				
Society Islands	47 (13–77)	8/18 (44 [26–69])	10/18 (56 [33–79])	18/49 (37 [26–47])
Tuamotu Islands	39 (7–86)	12/22 (55 [34–75])	10/22 (45 [25–66])	22/49 (45 [38–52])
Marquesas Islands	45 (10–82)	16/28 (57 [39–75])	12/28 (43 [24–61])	28/49 (57 [47–68])
Austral–Gambier Islands	38 (7–84)	19/29 (66 [48–83])	10/29 (34 [17–52])	29/49 (59 [39–80])
Total	41 (7–86)	55/97 (57 [47–67])	42/97 (43 [33–53])	97/196 (49 [42–57])
September–November 2015				
Society Islands	43 (4–88)	73/154 (47 [40–55])	81/154 (53 [45-61])	154/700 (22 [16–28])
Schoolchildren				
May–June 2014				
Society Islands	11 (6–16)	221/312 (71 [66–76[)	91/312 (29 [24–34])	312/476 (66 [60–71])

*CIs were calculated taking into account the cluster sampling design (*9*) and using the Fisher exact test.

## Conclusions

During the October 2013–April 2014 Zika infection outbreak in French Polynesia, ≈11.5% of the population sought medical care for symptoms suggestive of Zika infection (*1*,*7*); however, serosurveys at the end of the outbreak showed a Zika virus seroprevalence rate of 49% (95% CI 42%–57%), suggesting that most infected persons did not seek medical care. The finding that 43% (95% CI 33%–53%) of the participants had Zika virus IgG without self-reported symptoms reflects an estimated asymptomatic to symptomatic ratio of 1:1. These results suggest that infected persons did not consult medical care staff because the infection was mild or asymptomatic, as previously described (*10*). Of the 5 French Polynesia archipelagos, the Society Islands had the lowest seroprevalence rate (37% [95% CI 26%–47%]) and the Australs–Gambier Islands the highest (59% [95% CI 39%–80%]); however, seroprevalence among the archipelagos did not differ substantially, suggesting that no matter their location, study participants had similar Zika virus transmission exposure.

Eighteen months after the end of the outbreak, the Zika virus seroprevalence rate and proportion of asymptomatic infections among 700 persons on the Society Islands were 22% (95% CI 16%–28%) and 53% (95% CI 45%–61%), respectively, not substantially different from those during the first cluster sampling in the same islands (37% [95% CI 26%–47%] and 56% [95% CI 33%–79%], respectively). The finding that the Zika virus seroprevalence rate did not increase between the 2 sampling periods suggests that the virus did not actively circulate after the end of the outbreak. In contrast, the decrease in the Zika virus seroprevalence rate, even if not significant, suggests that Zika virus IgG titers may drop over time.

Within 2 months after the end of the outbreak, the Zika virus seroprevalence rate among schoolchildren (6–16 [median 11] years of age) on Tahiti was substantially higher than that among the general population (4–88 [median 43] years of age) (66% [95% CI 60%–71%] vs. 22% [95% CI 16%–28%], respectively). In contrast, the proportion of asymptomatic Zika virus infections was substantially lower among schoolchildren (29% [95% CI 24%–34%]) than among the general population (53% [95% CI 45%–61%]). Dengue virus (DENV) may provide cross-protection against Zika infection; thus, the higher DENV IgG seroprevalence among adults may explain why fewer adults than children were infected by Zika virus (*8*,*11*–*13*). The lower asymptomatic rate in children may have 2 additional explanations: the reporting of symptoms among children may have been compounded by the relatively higher frequency of febrile rash illness due to other viral infections, and sampling among children was conducted at the tail end of the outbreak, so they would likely remember symptoms more clearly than the population surveyed 18 months after the outbreak.

In the 3 groups tested, no difference was seen by sex in the seroprevalence rate or the proportion of asymptomatic infections. However, because the sampling scheme was not initially designed to compare data by sexes, data could not be extrapolated to the population level.

Our findings show that <50% of the population of French Polynesia had detectable Zika virus IgG. This seroprevalence rate is much lower than the 86% attack rate estimated by Kucharski et al. (*14*) using a model that assumed the French Polynesia population was 100% susceptible to Zika virus infection. However, in a setting where DENVs are highly prevalent (*8*), the possibility of cross-protecting immunity preventing infection from Zika virus (*12*,*13*) cannot be excluded. The attack rate and the asymptomatic:symptomatic ratio in French Polynesia were also lower than those described for the 2007 outbreak on Yap Island (73% and 4:1, respectively) (*4*); this finding supports the perception that the drivers of Zika virus transmission vary depending on geographic context. For other flaviviruses, such as DENV, previous model-based studies showed that the herd immunity threshold required to block viral transmission is ≈50%–85% (*15*). Thus, if Zika virus has the same epidemiologic characteristics as DENV, the seroprevalence rate of 49% would not be sufficient to prevent another outbreak.
